# RNA-Seq versus oligonucleotide array assessment of dose-dependent TCDD-elicited hepatic gene expression in mice

**DOI:** 10.1186/s12864-015-1527-z

**Published:** 2015-05-10

**Authors:** Rance Nault, Kelly A Fader, Tim Zacharewski

**Affiliations:** Department of Biochemistry and Molecular Biology, Center for Integrative Toxicology, Michigan State University, East Lansing, MI 48824 USA

**Keywords:** RNA-Seq, Microarray, Comparison, TCDD, Dose–response, Mouse, Liver

## Abstract

**Background:**

Dose-dependent differential gene expression provides critical information required for regulatory decision-making. The lower costs associated with RNA-Seq have made it the preferred technology for transcriptomic analysis. However, concordance between RNA-Seq and microarray analyses in dose response studies has not been adequately vetted.

**Results:**

We compared the hepatic transcriptome of C57BL/6 mice following gavage with sesame oil vehicle, 0.01, 0.03, 0.1, 0.3, 1, 3, 10, or 30 μg/kg TCDD every 4 days for 28 days using Illumina HiSeq RNA-Sequencing (RNA-Seq) and Agilent 4×44 K microarrays using the same normalization and analysis approach. RNA-Seq and microarray analysis identified a total of 18,063 and 16,403 genes, respectively, that were expressed in the liver. RNA-Seq analysis for differentially expressed genes (DEGs) varied dramatically depending on the P1(*t*) cut-off while microarray results varied more based on the fold change criteria, although responses strongly correlated. Verification by WaferGen SmartChip QRTPCR revealed that RNA-Seq had a false discovery rate of 24% compared to 54% for microarray analysis. Dose–response modeling of RNA-Seq and microarray data demonstrated similar point of departure (POD) and ED_50_ estimates for common DEGs.

**Conclusions:**

There was a strong correspondence between RNA-Seq and Agilent array transcriptome profiling when using the same samples and analysis strategy. However, RNA-Seq provided superior quantitative data, identifying more genes and DEGs, as well as qualitative information regarding identity and annotation for dose response modeling in support of regulatory decision-making.

**Electronic supplementary material:**

The online version of this article (doi:10.1186/s12864-015-1527-z) contains supplementary material, which is available to authorized users.

## Background

Toxicogenomic evaluations by microarrays have been invaluable in elucidating underlying mechanisms of toxicity [1,2], investigating species-specific responses and ligand potencies [3-7], and linking differential gene expression to apical endpoints [8,9]. However, the emergence of next-generation-sequencing (NGS) with its direct transcript identification, lower cost, larger dynamic range and superior detection of low abundance genes [10-12], is making microarrays obsolete. Despite these advantages, some studies have raised concerns regarding RNA-Seq and microarray comparability [13-15]. Probe design and differences in platform sensitivity have been offered as potential explanations, although normalization and analysis methods also contribute to low concordance [1,13,16].

Analysis approaches have been developed specifically for RNA-Seq [17-21], yet repurposed microarray normalization, statistical analysis, and DEG identification methods outperform several RNA-Seq specific analysis tools [20,21]. Consequently, we investigated the use of our semi-parametric normalization [22] and empirical Bayes analysis approach [23], traditionally used for microarrays. Semi-parametric normalization accounts for multiple sources of variation including random effects [22] while empirical Bayes analysis has the advantage of considering continuous variables such as the correlation between doses that can improve DEG detection [23,24], an important consideration in regulatory decision-making.

While microarray data submissions are encouraged by regulatory agencies and widely accepted in toxicogenomic research [2,13], the third phase of the microarray quality control project (MAQC-III also known as SEQC) recommended further validation using different study designs [1,25]. To date, study designs involving chemical treatment have been limited to single dose studies [1,15], the impact of different normalization approaches, or dose–response RNA-Seq studies with a sequencing depth of only 5 M reads [13]. Collectively, these studies reported comparable responses between platforms, but were mixed in the ability of RNA-Seq to reveal the biological relevance of responses.

2,3,7,8-Tetrachlorodibenzo-*p*-dioxin (TCDD) is a persistent environmental contaminant that binds to the aryl hydrocarbon receptor (AhR) which then translocates to the nucleus and dimerizes with the aryl hydrocarbon receptor translocator (ARNT) [26,27]. The TCDD-AhR-ARNT complex then binds to regulatory regions and elicits changes in global gene expression using dioxin response element (DRE)-dependent [26,27], and -independent mechanisms [28-31]. Dose-and time-dependent TCDD elicited differential gene expression has been evaluated using microarrays after a single dose [7,9,32,33]. In this study, we extend these results by examining the effects of continuous TCDD exposure. Dose-dependent hepatic differential gene expression in mice following oral gavage with TCDD every 4 days for 28 days was compared using RNA-Seq and Agilent oligonucleotide microarrays analyzed using same the same normalization and empirical Bayes analysis.

Overall, RNA-Seq and Agilent generated comparable results. Quantitatively, RNA-Seq detected more genes expressed in the liver, and identified more differentially expressed genes (DEGs) compared to Agilent. Qualitatively, direct sequencing by RNA-Seq provided more accurate transcript identification. Verification by WaferGen SmartChip QRTPCR indicates RNA-Seq had fewer false-positives and false-negatives compared to Agilent. Dose response modeling was also consistent between both platforms, but the ability of RNA-Seq to detect low abundance transcripts and its larger dynamic range provided superior qualitative and quantitative data for DEG identification, and estimates of point of departure (POD) and ED_50_.

## Results

### Quantitative RNA-Seq advantages

**Figure 1:**
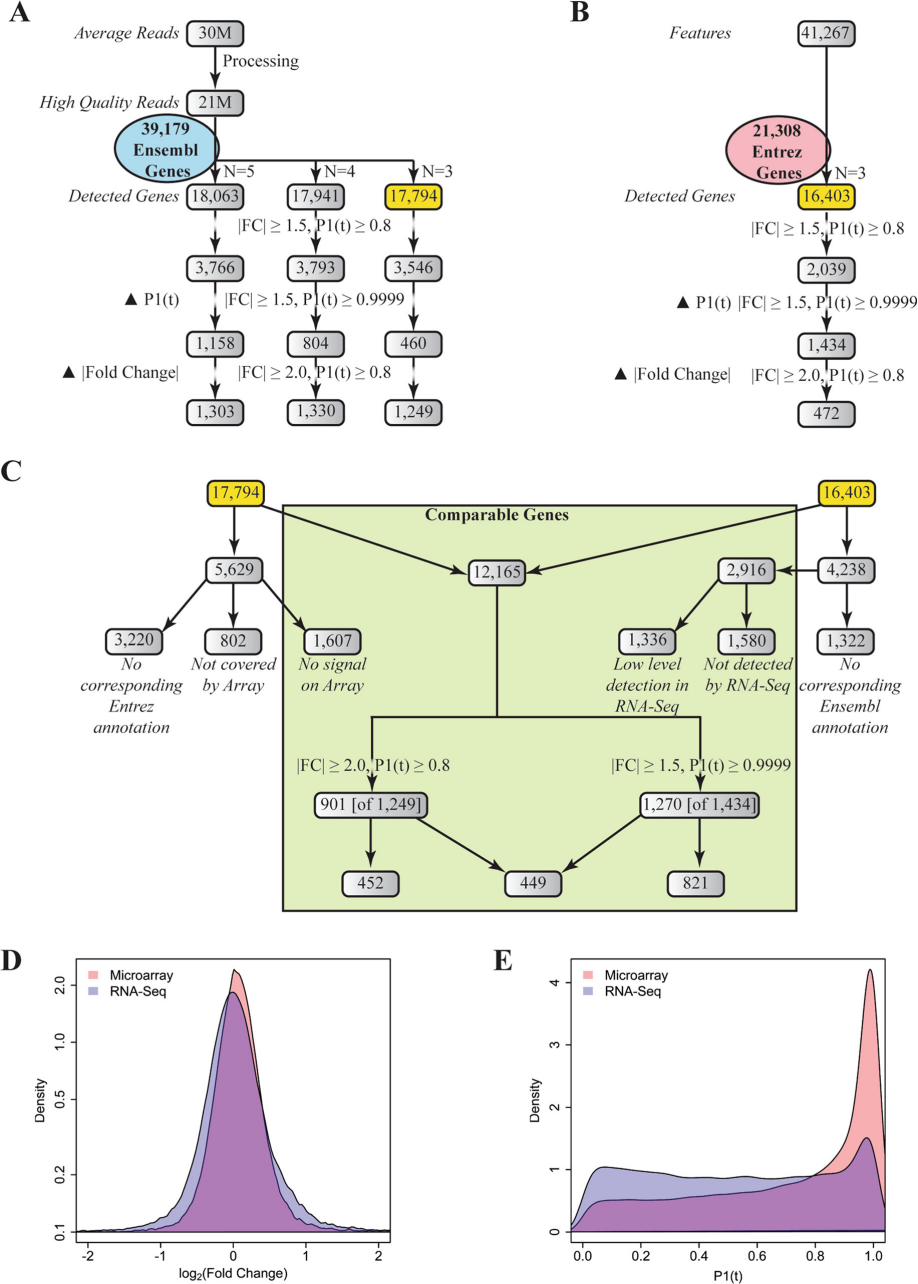
Comparing RNA-Seq and Agilent microarrays for detecting genes expressed in the liver and differentially expressed by TCDD. **(A)** RNA-Seq reads were aligned to mouse genome GRCm38 (release 74) and subsampled to represent 3–5 independent biological replicates. The number of differentially expressed genes (DEGs) was determined under varying |fold change| and P1(*t*) criteria. **(B)** Microarray features were examined for DEGs under varying |fold change| and P1(*t*) criteria. **(C)** RNA-Seq and microarray detected genes (yellow boxes) were examined for common and unique detected genes and DEGs. **(D)** Distribution of log_2_ (fold change) and **(E)** P1(*t*) values (P1 (*t*) ≥ 0) in RNA-Seq (blue) and Agilent (pink) datasets.

Genome-wide hepatic gene expression was examined using NGS Sequencing and Agilent 4×44 K oligonucleotide microarrays using the same RNA samples. RNA-Seq was performed at an average read depth of 30 M resulting in ~21 M high quality reads per sample (Figure 1A). Reads were mapped to the mouse reference genome GRCm38 (release 74) representing 39,179 Ensembl annotated genes. Genes with greater than 4 aligned reads in any sample were considered “expressed” or “detected”. Using this criteria ≥85% of expressed genes were present in all samples (Additional file 1). In total, 17,794 genes were found to be expressed in the mouse liver with a sample size of 3. The number of genes expressed in the liver did not change appreciably between 3, 4, or 5 (17,794, 17,941 and 18,063, respectively) biological replicates (Figure 1A). Conversely, Agilent microarrays have 41,267 predefined probes representing 21,308 unique Entrez annotated genes (Figure 1B). A total of 16,403 genes were “detected” (expressed in the liver) based on the median feature intensity being greater than the median background intensity as determined by GenePix.

Comparison of detected genes using three biological replicates identified 12,165 genes which were detected either by RNA-Seq or microarray and annotated with corresponding Entrez and Ensembl Gene IDs (Figure 1C). Among the 5,629 genes detected only by RNA-Seq, 1,607 were represented on the microarray but not detected based on GenePix’s signal to background ratio. Similarly, 2,916 genes of the 4,238 detected by microarray only were not detected by RNA-Seq when using a count cut-off of greater than 4 aligned reads. However 1,336 of these 2,916 genes did have at least 1 read align in at least one sample while the remaining 1,580 were not detected by RNA-Seq. In addition, 1,322 and 3,202 genes detected by RNA-Seq and microarrays, respectively, did not have corresponding Entrez or Ensembl annotation that largely represented predicted genes (i.e. ENSMUSG00000099065; Gm19980 predicted gene 19980). Most importantly, there were 802 genes not covered by the microarray highlighting the quantitative benefit of RNA-Seq’s open concept platform.

### RNA-Seq dynamic range influences filtering criteria

Contrary to previous comparative studies, we used the same semi-parametric normalization and empirical Bayes analysis to identify DEGs in both RNA-Seq and microarray datasets. Empirical Bayes analysis allows the varying of fold change and posterior probability (P1(*t*)) cut-offs to investigate the effects of filtering criteria on DEG detection without violating parametric hypothesis testing assumptions.

**Figure 2:**
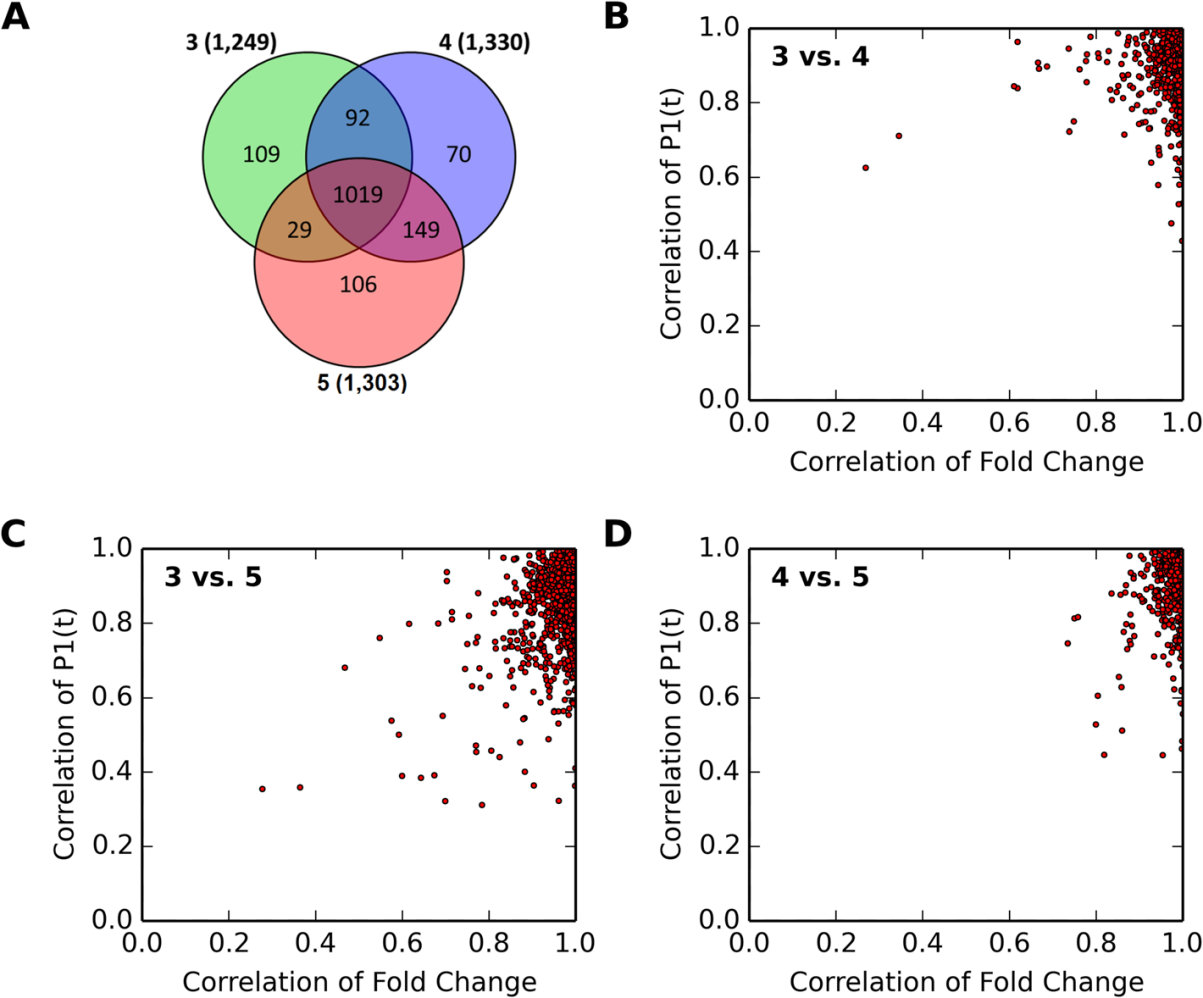
Effect of the number of independent biological replicates on RNA-Seq analysis. **(A)** DEGs were identified using a |fold change| ≥ 2.0 and P1(*t*) ≥ 0.8 when examining 5 independent biological replicates or a subset of 3 or 4 replicates and compared for identified genes. Correlation of gene expression fold changes and P1(*t*) values are illustrated comparing **(B)** 3 and 4, **(C)** 3 and 5, and **(D)** 4 and 5 biological replicates.

The number of identified DEGs did not change appreciably between 3, 4, or 5 biological replicates (Figure 1A) with ~78% (1,019 genes) identified in all three sample size subsets (Figure 2A). The other ~22% were also expressed in the other datasets but did not meet the filtering criteria. Relaxation of filtering criteria within the union increased the overlap to ~92%, and all of these genes exhibited a positive fold change and P1(*t*) value correlation (all found in upper right quadrant; Figure 2B-D). Given the excellent correspondence of DEG responses across all sample sizes, all subsequent analyses were performed using three independent biological replicates representing the same samples as the RNA-Seq analysis to facilitate fairer comparisons to the microarray data set which also used three biological replicates.

RNA-Seq data was found to be more sensitive to changes in the P1(*t*) filtering criteria. Increasing the P1(*t*) cut-off from ≥0.8 to ≥0.9999 while maintaining a |fold change| cut-off of ≥1.5 decreased the number of DEGs from 3,546 to 460 whereas increasing the |fold change| cut-off from >1.5 to >2.0 decreased the number of DEGs from 3,546 to 1,249 (Figure 1A). Conversely, for the microarray analysis, increasing the |fold change| cut-off from >1.5 to >2.0 dramatically decreased the identification of DEGs from 2,039 to 472 while increasing the P1(t) cut-off had a less dramatic effect (2,039 DEGs at |fold change| ≥ 1.5, P1(t) ≥0.8 compared to 1,434 DEGs at |fold change| ≥ 1.5, P1(t) ≥0.9999) (Figure 1B).

These differences in sensitivity to filtering criteria may be attributed to the larger dynamic range of RNA-Seq which exhibits a wider range of fold changes (Figure 1D) and a more uniform distribution of P1 (*t*) values (Figure 1E). Consequently, a |fold change| ≥ 2.0 and P1(*t*) ≥ 0.8 were used for RNA-Seq while a |fold change| ≥ 1.5 and P1(*t*) ≥ 0.999 were used for microarray in all subsequent comparisons in this study.

### Comparison of RNA-Seq and Agilent datasets

Comparing RNA-Seq and Agilent datasets identified 12,165 genes commonly expressed in the liver (Figure 1C). Genes showing the strongest responses in both RNA-Seq and microarray datasets included the typical AhR responsive genes such as *Cyp1a1*, *Cyp1a2*, *Nqo1*, and *Tiparp* (Additional file 2). *Cyp1a1* exhibited a 771- and 82-fold induction, while *Sult3a1* was repressed 100- and 25- fold by RNA-Seq and microarray analysis, respectively. The magnitude of the fold change at both extremes illustrate the difference in dynamic range between the two platforms.

**Figure 3:**
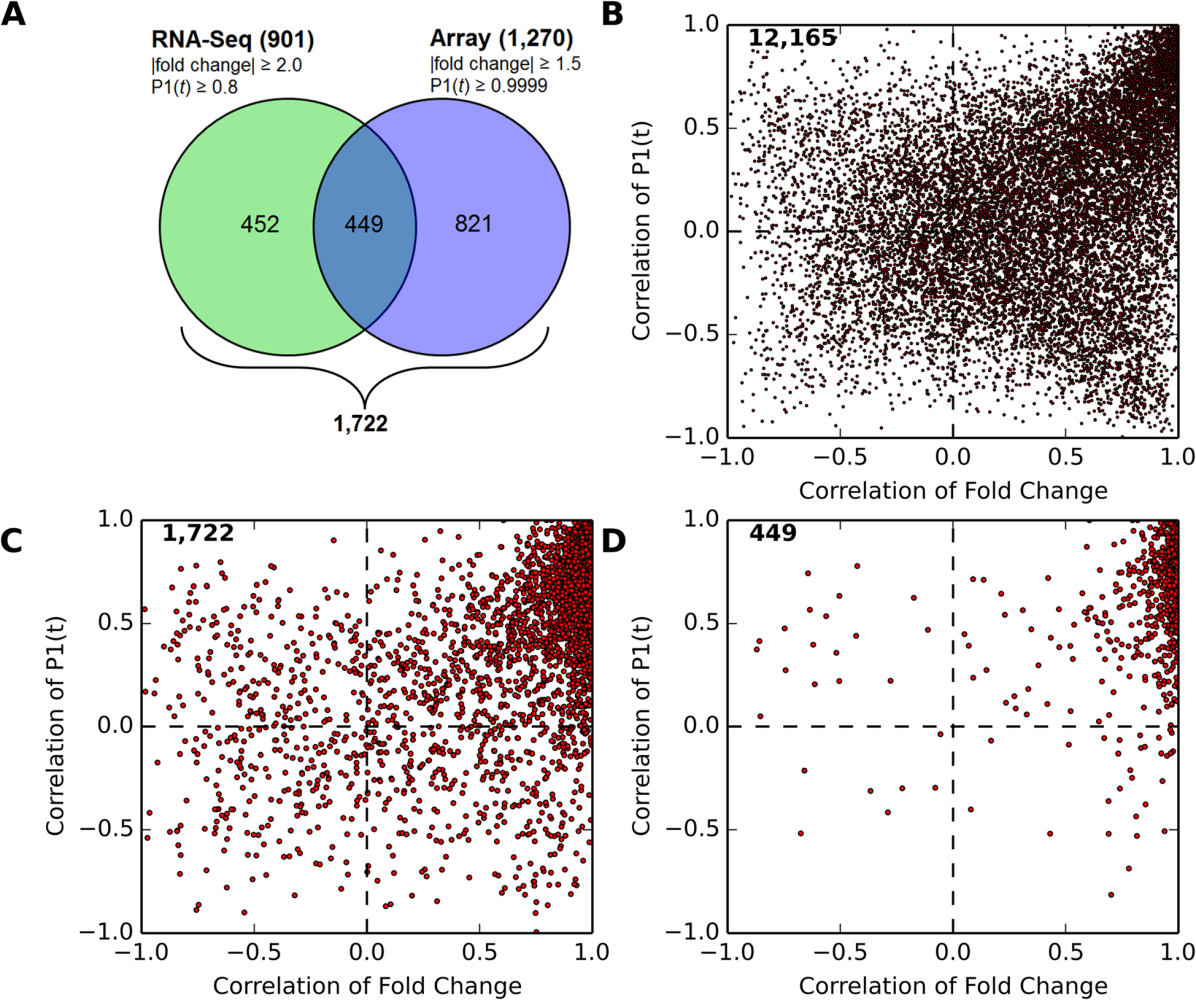
Comparison of RNA-Seq and Agilent DEGs. **(A)** Overlapping, RNA-Seq-specific and Agilent-specific DEGs were identified. Correlation of fold changes and P1(*t*) values were examined for **(B)** all 12,165 expressed genes detected by both platforms, **(C)** the union of 1,722 differentially expressed genes (DEGs), and **(D)** the 449 DEGs detected by both platforms.

Within the 12,165 common RNA-Seq and microarray genes, 901 and 1,270 DEGs were identified, respectively (Figure 1C). Comparative analysis revealed that only 449 genes were common to both datasets (Figure 3A) and that a similar overlap is observed for each dose independently (data now shown). Nevertheless, a majority of the 12,165 genes detected by both platforms (Figure 3B), the union of 1,722 DEGs across both platforms (Figure 3C), and the 449 DEGS common to both platforms (Figure 3D) exhibited positive fold change and P1(*t*) value correlations, indicating a strong correlation between RNA-Seq and Agilent. As seen with sample size, relaxing the cut-off criteria for the union of the RNA-Seq and microarray data sets increased the overlap suggesting differences were most likely due to genes approaching but not satisfying hard fold change and/or P1(*t*) cut-offs, despite evidence of differential expression.

Despite the overlap of only 449 genes, comparison of functional enrichment analyses identified DEGs associated with lipid binding, processing, and metabolism, oxidative stress, immune responses, cell adhesion and movement, and extracellular matrix remodeling within RNA-Seq and Agilent data sets (Additional file 3: Table S2) consistent with previous reports [32,34]. Additional analysis of genes unique to RNA-Seq also identified carbohydrate binding and extracellular matrix remodeling as enriched functions while unique microarray genes show lower enrichment of these functions (Additional file 4: Table S3).

### RNA-Seq outperforms microarray DEG identification

**Figure 4:**
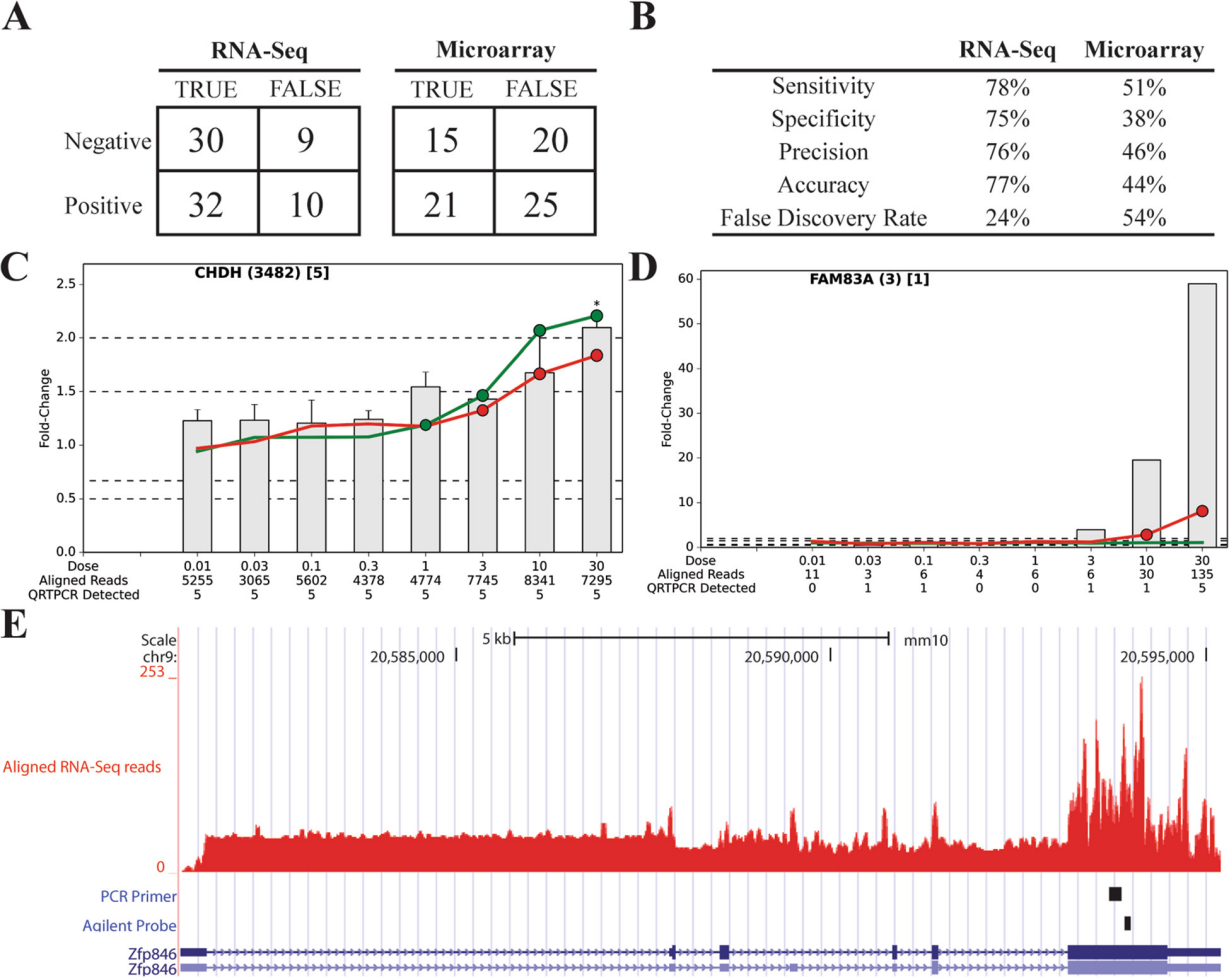
Verification of RNA-Seq and Agilent DEG identification by WaferGen SmartChip Real-Time PCR analysis. **(A)** QRTPCR data (n = 5) was used as the “gold standard” to determine true and false positives and negatives for RNA-Seq (n = 3) and Agilent (n = 3) datasets. **(B)** Performance metrics of RNA-Seq and Agilent validated by QRTPCR. **(C)** Representative example of a false-negative and **(D)** false-positive response in the RNA-Seq dataset. Official gene symbols are indicated in upper left corner with the number of RNA-Seq aligned reads in parentheses () and number of samples with C_t_ values lower than background in brackets [] for vehicle control samples. Bars represent mean fold-change determined by WaferGen technology (±SEM), the red line represents RNASeq fold-change, and the green line represents Agilent fold change. Significant differences within WaferGen data were determined by one-way ANOVA followed by Dunnett’s *post-hoc* test and indicated by an asterisk (*) with the exception of *Fam83a* whose undetectable levels prevented statistical testing. Red (RNA-Seq) and green (Agilent) dots represent P1 (*t*) values with size indicating level of significance (small ~0.8, large ~1). Labels on the X-axis indicate the dose of TCDD (μg/kg), number of aligned RNA-Seq reads, and number of samples with C_t_ values lower than background. Dashed lines indicate 1.5 and 2.0 |fold-change| thresholds to identify DEGs. **(E)** UCSC genome browser track illustrating PCR primer and Agilent probe alignments for Zfp846. The *Zfp846* loci is presented in blue with exons (closed boxes) and introns (solid line). The arrowheads indicate the direction of transcription.

A subset of RNA-Seq and Agilent responses were further investigated using WaferGen SmartChip Real-Time PCR. This included 7 negative controls (unchanged in both datasets), 13 positive controls (changed in both datasets), 35 RNA-Seq specific genes, and 34 Agilent specific genes (Additional file 5). The platform-specific genes included 20 exhibiting divergent responses (maximum of |RNA-Seq fold change – microarray fold change|). Of the 81 genes validated, Agilent identified 20 false negatives and 25 false positives for a false discovery rate (FDR) of 54% while RNA-Seq found 9 false-negatives and 10 false-positives for a FDR of 24% (Figure 4A). Overall, RNA-Seq outperformed Agilent on sensitivity, specificity, precision, accuracy, FDR, and false negative rate (Figure 4B).

Further examination of false negatives/positives identified by RNA-Seq revealed that false negatives were largely due to failing to meet the filtering criteria, despite remarkably similar expression patterns across all three technologies (Figure 4C and Additional file 6). False positives typically included genes with low numbers of reads and, in most cases, changed expression from non-detectable to modest or vice-versa, suggesting they are in fact true positives (Figure 4D and Additional file 7).

Agilent genes showing the most divergent expression compared to WaferGen were *Disp2*, *Gabbr1*, *Heatr5a*, *Wdr11*, *Zfp524*, and *Zfp846* (Additional file 8), while *Sult2a7*, *Tlr5*, and *Uox* were only identified as DEGs by RNA-Seq (Additional file 9). Overall, there was excellent agreement between RNA-Seq and WaferGen with Agilent exhibiting divergence. Examination of Agilent probes revealed *Disp2* and *Tlr5* did not match their intended target, whereas *Heat5ra* and *Wdr11* exhibited different responses depending on the Agilent probes querying different gene regions. However, the Agilent probe sequence and WaferGen primers for *Zfp846* queried adjacent regions suggesting non-specific hybridization (Figure 4E). Therefore, in addition to artifactual platform specific differential gene expression due to hard cut-off criteria, discrepancies between RNA-Seq and Agilent can also be attributed to differences in sensitivity and probe designs that query different regions, have no known target (mis-annotated) and/or are susceptible to cross-hybridization.

### RNA-Seq versus Agilent dose response analysis

**Figure 5:**
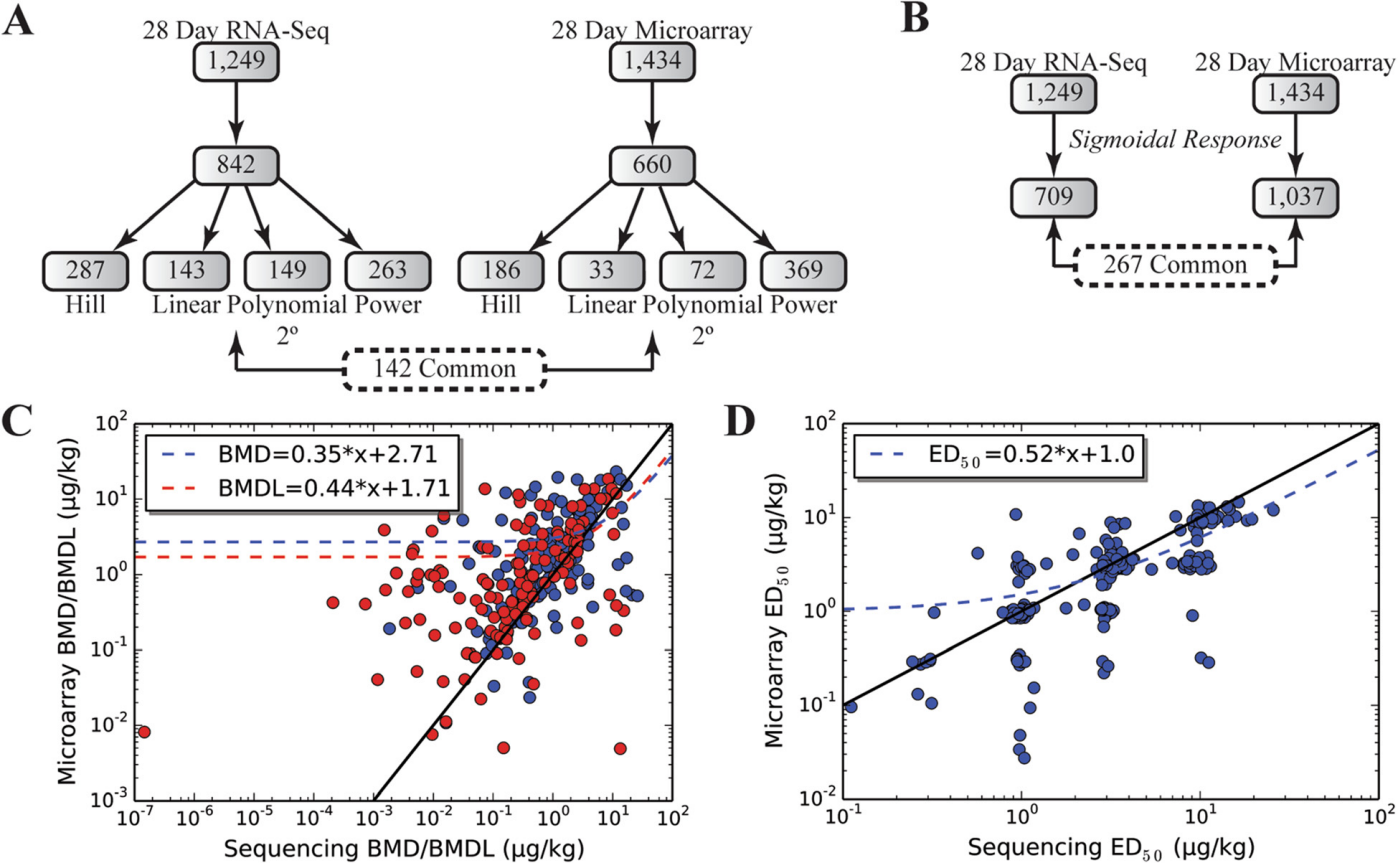
Comparison of RNA-Seq and Agilent dose–response modeling. **(A)** The ToxResponse modeler [36] was used to identify genes fitting a sigmoidal response for the estimation of ED_50_s while **(B)** BMDExpress [35] was used to find the best fit curves for point of departure (BMD and BMDL) estimates. Correlation of **(C)** ED_50_s and **(D)** BMD (blue circles) and BMDL (red circles) estimates were examined for all comparable genes.

Dose–response modeling is critical in regulatory decision-making. Consequently, point of departure (POD) estimates for the benchmark dose (BMD; dose at which response begins to be different from control) and the benchmark dose limit (BMD(L); lower limit of a one-sided 95% confidence interval on the BMD) were determined using BMDExpress [35]. BMDExpress fit 842 of 1,249 RNA-Seq DEGs with dose response curves compared to 660 of 1,434 Agilent DEGs (Figure 5A). Within the 449 DEGs common to both technologies, 142 (32%) were fitted with dose–response curves. Platform difference in dynamic range and ability to detect low abundant transcripts influenced POD estimates. For example, at low BMD(L) estimates, Agilent values were higher compared to RNA-Seq due to poorer resolution at low expression levels. At higher BMD(L) estimates RNA-Seq values were higher, likely due to microarray signal compression (Figure 5C). Nevertheless, POD estimates and rank were strongly correlated between both technologies (Figure 5C and Additional file 10). Moreover, POD estimates were not influenced by sample size (data now shown).

For ED_50_ estimates, 709 of 1,249 RNA-Seq DEGs exhibited a sigmoidal dose response as determined by the ToxResponse Modeler [36] while 1,037 of 1,434 Agilent DEGs exhibited a sigmoidal dose response with 267 in common between both platforms (Figure 5B). Correlation of the ED_50_s (dose at which the response is 50% of maximal response), found Agilent ED_50_s to be higher compared to RNA-Seq at low estimates. In contrast, RNA-Seq ED_50_s were higher at higher estimates (Figure 5C). The lower slope (0.52) is consistent with microarray signal compression and poorer ability to detect low abundance transcripts.

## Discussion

Whole genome gene expression analysis provides comprehensive data on potential mechanisms of action as well as product safety [1,2]. As regulatory agencies and researchers struggle to incorporate omic data into decision-making and research applications, the technology continues to evolve requiring the verification of reproducibility, reliability, and continuity [16,25]. Hybridization-based platforms such as Affymetrix GeneChips and Agilent oligonucleotide microarrays are being replaced by NGS technologies such as RNA-Seq. Although several studies have reported comparable technical reproducibility, variance structure, absolute expression, and DEG identification capabilities using different study designs and model systems [1,11,14,15,25], only one has investigated dose response [30], the corner stone of risk/safety assessment. Our study complements and extends previous comparisons by evaluating the dose-dependent hepatic changes in gene expression using RNA-Seq and Agilent 4×44 K oligonucleotide microarrays. We used the same normalization and analysis methods to minimize bias while further investigating the cause of qualitative and quantitative differences between RNA-Seq and Agilent oligonucleotide microarrays. In comparison to WaferGen SmartChip QRTPCR, we demonstrate that some differences between RNA-Seq and Agilent DEG identification may not be as significant as previously reported.

DEGs were identified using an adapted semi-parametric normalization approach followed by an empirical Bayes method that used model-based t values to calculate posterior probability P1(*t*) values on a per gene and dose basis [22,23]. Bayes models take into account adjacent points to consider trends within time course and dose response data sets. Therefore, P1(*t*) values do not test hypotheses, and can be used to rank and prioritize DEGs based on their probability of differential expression. This allows cut-off criteria to be varied in order to include well characterized responsive genes and mechanistic significance in the context of observed phenotypes, without violating any assumptions. In our study, varying the cut-off criteria demonstrated that RNA-Seq was particularly sensitive to P1(*t*) changes likely due to its much larger dynamic range compared to microarrays (Figure 1A, E).

The influence of sample size on DEG identification has not been adequately investigated. Power analyses suggest sample sizes of 3–25 depending on sequencing depth and budget [37] while others have examined 2, 4, and 5 replicates using synthetic datasets and various analysis approaches [20]. We identified comparable numbers of DEGs when using 3, 4, or 5 independent replicates at a sequencing depth of 30 M (Figure 2A). Moreover, there was a strong correlation across all sample sizes indicating RNA-Seq responses were not significantly influenced by the sample size (Figure 2B-D), facilitating cross-technology comparisons using a sample size of 3 in this study.

Overall, RNA-Seq detected more genes expressed in the liver (Figure 1A). Many of the genes only detected on Agilent microarrays could also be identified within the RNA-Seq dataset by lowering the number of reads threshold (Figure 1C). Similarly, most DEGs identified only on Agilent microarrays could also be detected in the RNA-Seq dataset by lowering the selection criteria, particularly the P1(*t*) cut-off. Comparing RNA-Seq and Agilent datasets for DEGs identified a 35% – 50% overlap (Figure 3A), similar to previous reports [1,13,14]. WaferGen QRTPCR, RNA-Seq and Agilent dose response curves were remarkably similar for many genes classified as either RNA-Seq- or Agilent-specific (Figure 4C, D and Additional file 7: Figure S4, Additional file 8: Figure S5, Additional file 9: Figure S6, Additional file 10: Figures S7) suggesting that differences between platforms were due more to selection criteria as opposed to differences in expression. Consequently, RNA-Seq quantitatively outperformed Agilent microarrays when identifying the total number of genes expressed in the liver, and differentially expressed by TCDD (Figure 4B). In addition, RNA-Seq provided more definitive qualitative data regarding the identity of the gene that the transcript represented and identified more genes involved in pathways known to be affected by TCDD. Previous studies suggest RNA-Seq performance was mixed for detecting the differential expression of low expressed genes [1,13]. However, detection of low abundance genes improves dramatically at ~30 M reads [1,13], the sequencing depth used in this study.

Further analysis of genes exhibiting divergent expression (e.g. upregulated in RNA-Seq and downregulated in microarray) identified two principal contributing factors. First, some Agilent probes were misannotated and did not target the expected gene possibly due to outdated annotation provided by manufacturer, but nevertheless, requiring re-evaluation and re-annotation. Second, multiple probes for the same gene also showed divergent responses indicating either non-specific binding (cross-hybridization) or the presence of variant transcripts that targeted individual probes. RNA-Seq analysis mitigates these disadvantages by using the most recent genome build available and its associated annotation, while providing the opportunity to identify treatment-specific transcript variants.

Risk assessments typically involve four components: hazard identification, dose response characterization, exposure assessment and risk characterization. Omic technologies are expected to improve risk assessment by providing more qualitative and quantitative data. This includes the use of gene expression profile or fingerprint classifiers that not only identify comparable modes/mechanisms of action in hazard identification, but could also justify the use of refined uncertainty factors for extrapolation between species. In addition, RNA-Seq provides a greater number of responsive genes that can be functionally annotated, associated with a key event, and modeled for ED_50_ values and point of departure (POD) estimates such as BMD and BMD(L). Despite the challenges of incorporating omic data into risk assessment, the goal is to provide data that complements existing testing guidelines and requirements to support a more quantitative, mechanistically-based risk assessment.

RNA-Seq and Agilent datasets were analyzed for TCDD-elicited dose-dependent gene expression using ToxResponse modeler and BMDExpress. Although only a small subset of genes exhibited dose-dependent responses in both technologies (Figure 5A, B), the correspondence between both was strong. Overall, RNA-Seq POD and ED_50_ values were considered to be more accurate due to its ability to detect lower abundance genes and greater dynamic range (Figures 1D, E and 5C, D). Data compression and lower sensitivity also affects the slope of the dose response curve which may confound interpretation of receptor interaction mechanisms and cross-ligand comparisons used to determine toxic equivalency factor (TEF) estimates for polychlorinated dibenzo-p-dioxins (PCDDs), dibenzofurans (PCDFs), and dioxin-like polychlorinated biphenyls (PCBs) [38].

## Conclusions

RNA-Seq exhibited superior qualitative and quantitative performance compared to Agilent microarrays. Our results are not only consistent with most, if not all, previous comparative studies [1,12-14], but also provide additional complementary dose response evidence in a mouse model. Furthermore, differences between RNA-Seq and Agilent differential gene expression are largely due to filtering criteria and gene annotation, not differences in patterns of expression. Although some results between the platforms are comparable, accumulating evidence supports the use of RNA-Seq over microarrays for dose response studies.

## Methods

### Animal handling and treatment

Female C57BL/6 mice from Charles Rivers Laboratories (Portage, MI) were received on postnatal day 25 (PND25), housed in polycarbonate cages with cellulose fiber chips (Aspen Chip Laboratory Bedding, Northeastern Products, Warrensburg, NY) at 30–40% humidity, and acclimatized for 4 days using a 12 h light/dark cycle. Mice were fed *ad libitum* with Harlan Teklad 22/5 Rodent Diet 8940 (Madison, WI) and had free access to deionized water. On PND 28 and every following 4th day animals (N = 5) randomly assigned to treatment groups were orally gavaged with 0.1 mL sesame oil vehicle control or 0.01, 0.03, 0.1, 0.3, 1, 3, 10, or 30 μg/kg TCDD (Dow Chemical Company, Midland, MI) for a total of 28 days. On day 28 mice were sacrificed and livers were frozen in liquid nitrogen, and stored at-80°C. We assumed that randomization and consistent conditions across all treatment groups negated any gene expression effects due to differences in estrous stage. All procedures were carried out with the approval of the Michigan State University All-University Committee on Animal Use and Care.

### RNA isolation

Frozen liver samples (~100 mg) were transferred to 1.3 mL of TRIzol (Life Technologies, Carlsbad, CA) and homogenized using a Mixer Mill 300 tissue homogenizer (Retsch, Germany). Total RNA was isolated according to manufacturer’s protocol with an additional phenol:chloroform extraction (Sigma-Aldrich, St. Louis, MO). Isolated RNA was resuspended in RNA storage solution (Life Technologies). Total RNA was quantified and assessed for purity by nanodrop (Thermo Scientific, Waltham, MA), Qubit (Life Technologies), and Bioanalyzer (Agilent Technologies, Santa Clara, CA). Total RNA quality was also assessed using the A_260_/A_280_ ratio and by visual inspection on a denaturing gel. All sample processing and analysis was performed blinded to treatment group when possible.

### Microarray study design

Total RNA from treated animals were hybridized with vehicle control samples to 4×44 K Agilent microarrays (version 1; Agilent Technologies, Inc.). Microarrays were performed with three biological replicates (N = 3), commonly used in microarray assessments [9,32,33], using two independent labelings (Cy3 and Cy5) with dye-swap according to manufacturer’s protocol (Agilent Manual: G4140–90040 v. 5.7). Microarray slides were scanned at 532 nm (Cy3) and 635 nm (Cy5) on a GenePix 4000B scanner (Molecular Devices, Sunnyvale, CA). Images were analyzed for feature and background (circular region with a 3x larger diameter around the feature) intensities using GenePix Pro 6.0 (Molecular Devices). GenePix called a feature “detected” (i.e., expressed in the liver) when the median feature intensity was greater than the median background intensity.

Data were normalized using a semi-parametric approach [22] in SAS v9.3 (SAS Institute Inc., Cary, NC). Posterior probabilities (P1(*t*)) values were calculated using an empirical Bayes method based on a per gene and dose basis using model-based t values [23]. Priors were estimated using model-based t-values calculated for all probes and doses. Specifically, the empirical distribution of f (t, dose) is estimated from the 371,403 t-values (41,267 probes × 9 doses). The data was annotated by NCBI Entrez Gene ID provided by manufacturer and managed in TIMS dbZach data management system [39] and deposited in the Gene Expression Omnibus database (GEO; GSE62903).

### RNA-Sequencing, alignment, and analysis

RNA-Sequencing was performed at the Michigan State University Research Technology Support Facility Genomics Core (RTSF, https://rtsf.natsci.msu.edu/genomics). In summary, libraries from five independent biological replicates (N = 5) were prepared using the Illumina TrueSeq RNA Sample Preparation Kit (Illumina, San Diego, CA) according to manufacturer’s instructions. Library sizes were confirmed using Caliper GX (Perkin Elmer, Waltham, MA), and quantified by qPCR using the Kapa Biosystems quantification kit (Wilmington, MA). Sequencing of libraries was performed by pooling 10 random samples at equimolar ratios, quantified again by qPCR (Kapa Biosystems), and distributed across two lanes of the flow cells to maintain library complexity, and loaded onto an Illumina HiSeq 2500 and clustered onboard.

The Michigan State High Performance Computer (MSU HPCC; https://icer.msu.edu/hpcc) was used for read processing and analysis. Reads, 1×50 bp with a seven-base index, were demultiplexed and quality was determined using FASTQC v0.11.2 (www.bioinformatics.babraham.ac.uk/projects/fastqc/). Adaptor sequences were removed using Cutadapt v1.4.1 [40] and low-complexity reads were cleaned using FASTX v0.0.14 (http://hannonlab.cshl.edu/fastx_toolkit/index.html). Reads were mapped to the mouse reference genome (GRCm38 release 74) using Bowtie 1.0.0 and TopHat v1.4.1 [41] using default paramaters and a minimum and maximum intron length of 10 and 15000, respectively. Alignments were converted to SAM format using SAMTools v0.1.19. (samtools.sourceforge.net/). Gene counts were determined using HTSeq v0.6.1 [42] in intersection-nonempty mode (−m intersection-nonempty). In this study, a gene was considered “detected” (i.e., expressed in the liver) when the number of aligned reads was greater than 4, which resulted in ≥85% of “detected” genes present in all samples. RNA-Seq data is deposited in GEO (GSE62903).

Counts were transformed through variance stabilizing transformation (VST) using the DESeq package [19] in R (www.r-project.org) according to the DESeq reference manual. Data was normalized using a semi-parametric approach [22] in SAS v9.3 (SAS Institute Inc., Cary, NC). Posterior probabilities P1(*t*) values were calculated using an empirical Bayes method based on a per gene and dose basis using model-based t values [23]. The priors were estimated using model-based t-values calculated for all detected genes and doses. For example, the empirical distribution of f (t, dose) for the n = 5 dataset is estimated from 162,567 t-values (18,063 genes × 9 doses).

### Functional enrichment analysis

Functional enrichment analysis was performed using the Database for Annotation, Visualization, and Integrated Discovery (DAVID, http://david.abcc.ncifcrf.gov) [43] filtered for gene ontology biological processes (BP), molecular functions (MF), and cellular component (CC). Functional categories were considered enriched when the –log scale geometric mean p-value ≤ 0.05 (enrichment score ≥ 1.3).

### WaferGen smartchip real-time PCR

Total RNA (2 μg) was reversed transcribed using SuperScript II (Invitrogen) and oligo-dT primers. The WaferGen SmartChip (WaferGen Biosystems, Fremont, CA) was prepared according to manufacturer’s instructions at a final cDNA concentration of 1.25 ng/μl, primer concentration of 250 nM, and 1X SYBR Green mastermix (Bio-Rad, Hercules, CA) dispensed using the WaferGen SmartChip Multisample Nanodispenser. Amplification (cycling conditions of 2.53 min at 95°C followed by 40 cycles of 34 sec at 95°C and 1.04 min at 60°C, followed by a melt curve) and detection was performed using the WaferGen Real-Time PCR Cycler at RTSF. Expression was determined using the 2^-ΔΔCt^ method standardized to the geometric mean of reference genes *ActB*, *B2m*, *Gapdh*, *Hmbs*, *Hprt*, *Rn18s*, and *Rps13*. Primer sequences are available in Additional file 5. Data were examined for normality and statistically tested by One-way ANOVA with dose as the factor, followed by Dunnett’s *post-hoc* test, which contrasts to vehicle control, using SAS 9.3.

### Dose–response modeling

Dose–response modeling for the estimation of ED_50_s was performed using the ToxResponse Modeler [36]. For RNA-Seq ED_50_ modeling, gene responses were split into groups of 50 and run in parallel on the MSU HPCC. Only genes with sigmoidal dose-responses (see Burgoon et al., 2008 [36] for example of a sigmoidal dose–response curve) were included in the estimation of ED_50_s. Point of departure (POD) estimates benchmark dose (BMD) and lower 95% confidence limit BMD (BMDL) were estimated as previously described using BMDExpress [8,35,44]. Briefly, gene signal intensities were fit to Hill, power, linear, and polynomial 2 models with a benchmark response factor of 1.349. Best-fit model selection was performed as previously described [8].

## Additional files

Additional file 1:
**Influence of minimum RNA-Seq aligned read threshold on inclusion of genes detected in all samples.**
Additional file 2:
**WaferGen SmartChip QRTPCR validation of typical AhR responsive genes.**
Additional file 3:
**DAVID functional enrichment analysis of DEGs.**
Additional file 4:
**DAVID functional enrichment analysis of technology specific DEGs.**
Additional file 5:
**WaferGen SmartChip QRTPCR primer sequences and validation results.**
Additional file 6:
**WaferGen SmartChip QRTPCR analysis of false-negative RNA-Seq genes.**
Additional file 7:
**WaferGen SmartChip QRTPCR analysis of false-positive RNA-Seq genes.**
Additional file 8:
**WaferGen SmartChip QRTPCR analysis of most divergent expression responses differentially expressed in Agilent microarray dataset.**
Additional file 9:
**WaferGen SmartChip QRTPCR analysis of most divergent expression responses differentially expressed in RNA-Seq dataset.**
Additional file 10:
**Rank order correlation of dose–response modelling estimates.**
