# Supplementary material for: RNA-Seq versus oligonucleotide array assessment of dose-dependent TCDD-elicited hepatic gene expression in mice
Source: BMC Genomics. 2015 May 10;16(1):373. doi: 10.1186/s12864-015-1527-z (PMC4456707; doi:10.1186/s12864-015-1527-z)
Supplement: Additional file 1: — Influence of minimum RNA-Seq aligned read threshold on inclusion of genes detected in all samples. [file 12864_2015_1527_MOESM1_ESM.pdf]

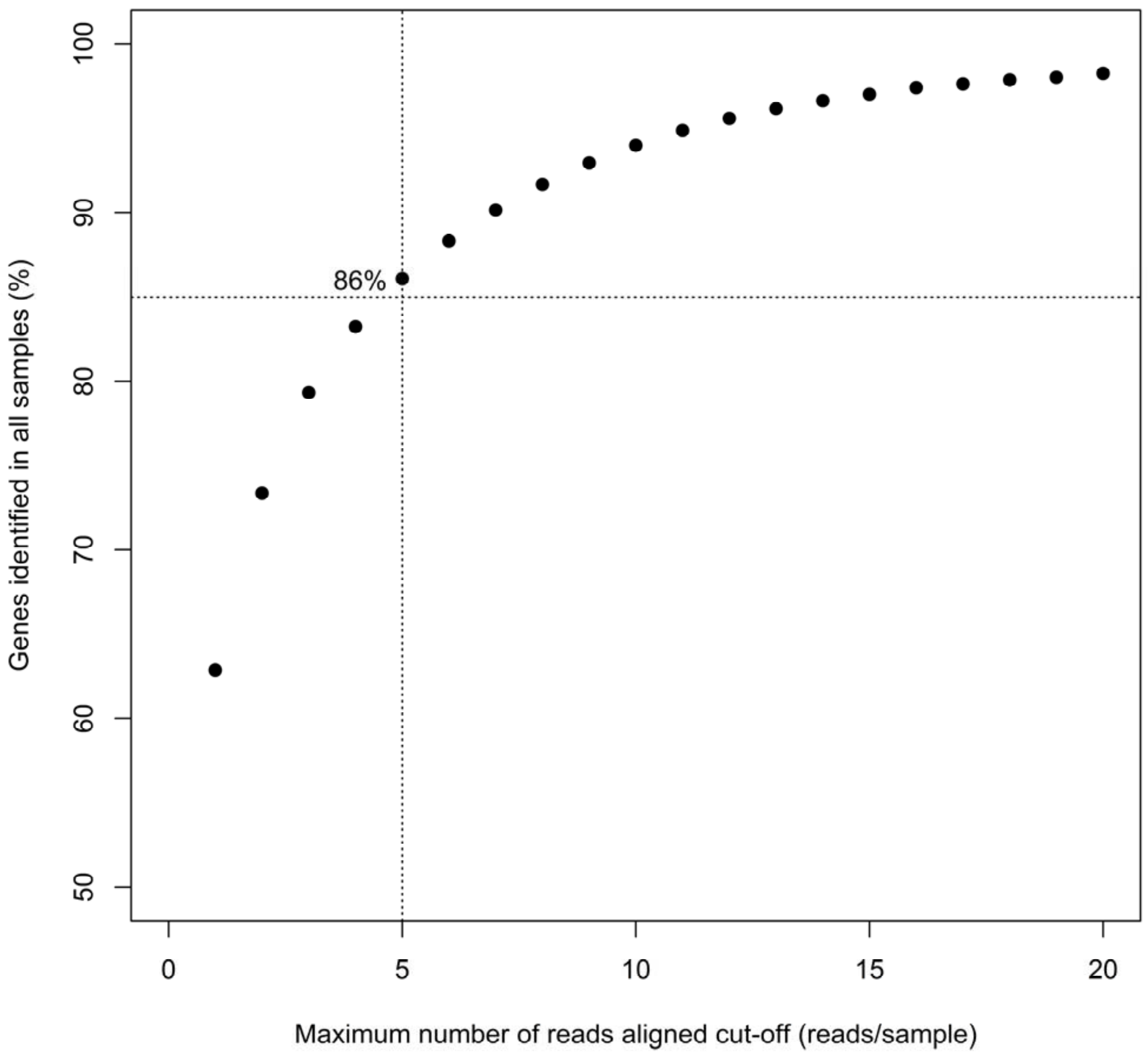

Supplementary Figure 1 Influence of minimum RNA-Seq aligned read threshold on the inclusion of genes detected in all samples. At a minimum of 5 aligned reads to call a gene "expressed or detected", 86% of genes included in the analysis were detected in all samples.
