# Supplementary material for: RNA-Seq versus oligonucleotide array assessment of dose-dependent TCDD-elicited hepatic gene expression in mice
Source: BMC Genomics. 2015 May 10;16(1):373. doi: 10.1186/s12864-015-1527-z (PMC4456707; doi:10.1186/s12864-015-1527-z)
Supplement: Additional file 9: — WaferGen SmartChip QRTPCR analysis of most divergent expression responses differentially expressed in RNA-Seq dataset. [file 12864_2015_1527_MOESM9_ESM.pdf]

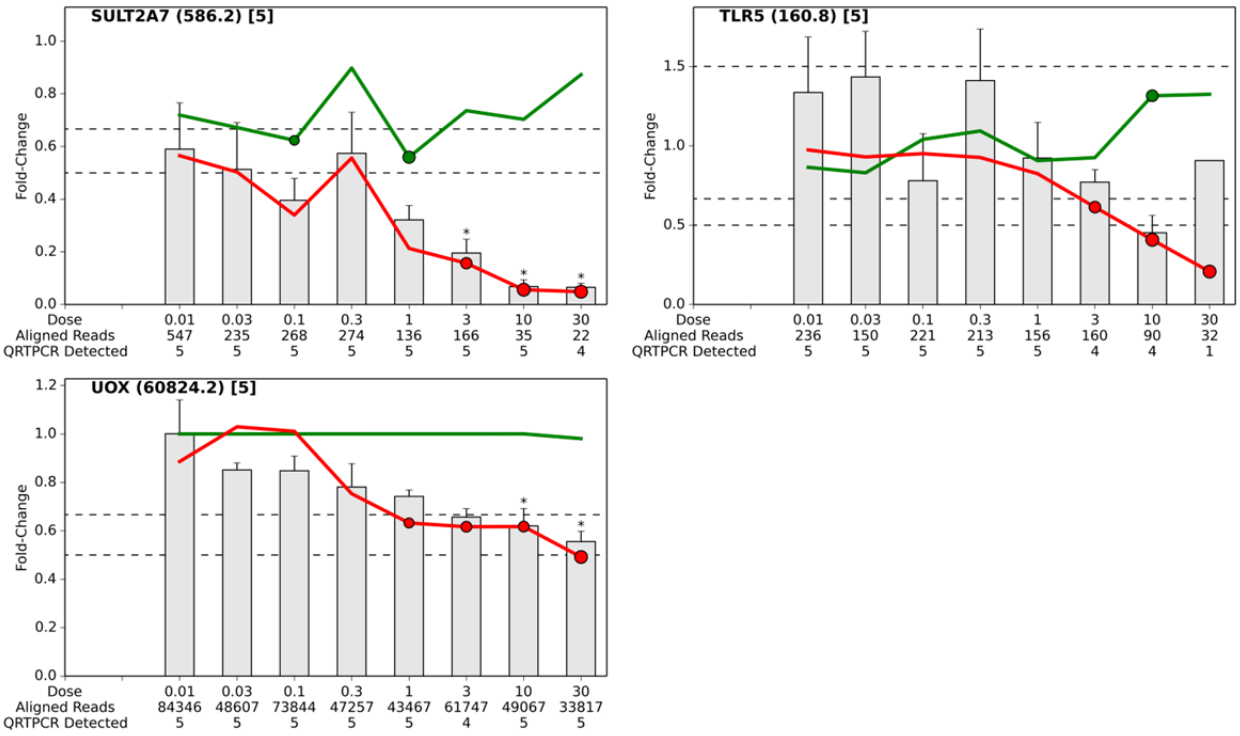

Supplementary Figure 6 WaferGen SmartChIP QRTPCR verification of the most divergent genes identified as DEG by RNA-Seq. Official gene symbols are indicated in upper left corner with the number of RNA-Seq aligned reads in parentheses ( ) and number of samples with C_t values lower than background in brackets [ ] for vehicle control samples. Bars represent mean fold-change determined by WaferGen technology (±SEM), the red line represents RNA-Seq fold-change, and the green line represents Agilent fold change. Significant differences within WaferGen data were determined by one-way ANOVA followed by Dunnett's *post-hoc* test and indicated by an asterisk (*). Red (RNA-Seq) and green (Agilent) dots represent P1(*t*) values with size indicating level of significance (small ~0.8, large ~1). Labels on the X-axis indicate the dose of TCDD (µg/kg), number of aligned RNA-Seq reads, and number of samples with C_t values lower than background. Dashed lines indicate 1.5 and 2.0 |fold-change| thresholds to identify DEGs.
