# Supplementary material for: RNA-Seq versus oligonucleotide array assessment of dose-dependent TCDD-elicited hepatic gene expression in mice
Source: BMC Genomics. 2015 May 10;16(1):373. doi: 10.1186/s12864-015-1527-z (PMC4456707; doi:10.1186/s12864-015-1527-z)
Supplement: Additional file 10: — Rank order correlation of dose–response modelling estimates. [file 12864_2015_1527_MOESM10_ESM.pdf]

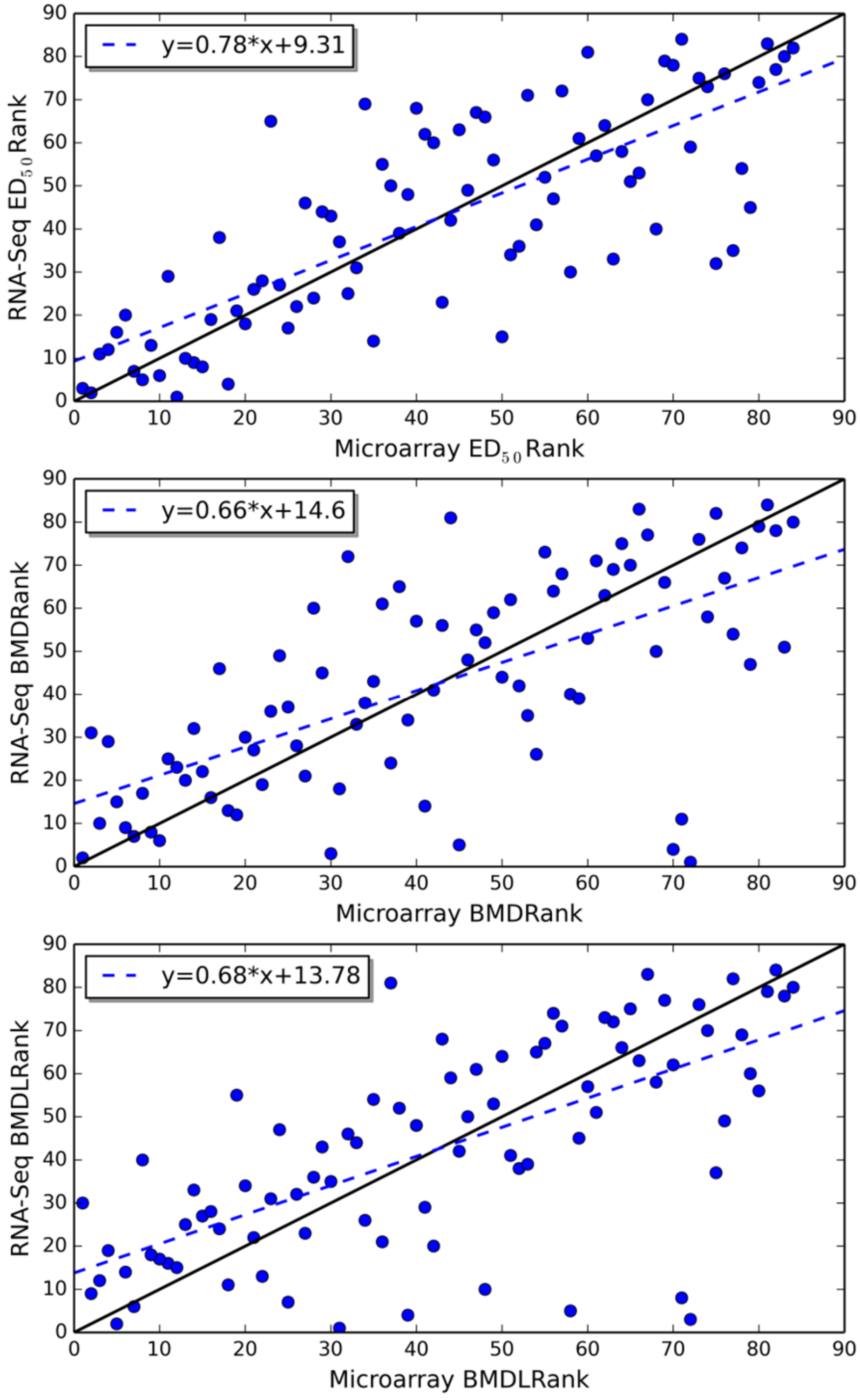

Supplementary Figure 7 Rank order correlation of dose-response modeling estimates. (**a**) bench mark dose limit (BMDL), (**b**) bench mark dose (BMD), and (**c**) effective dose eliciting a 50% response (ED_{50}s) were ranked in both RNA-Seq and Agilent datasets for the 267 genes determined to fit a sigmoidal response by ToxResponse modeler[34] or 142 genes with a best fit curve determined by BMDExpress[35].
